# Proteomics: a subcellular look at spermatozoa

**DOI:** 10.1186/1477-7827-9-36

**Published:** 2011-03-22

**Authors:** Stefan S du Plessis, Anthony H Kashou, David J Benjamin, Satya P Yadav, Ashok Agarwal

**Affiliations:** 1 Division of Medical Physiology, Stellenbosch University, Tygerberg, South Africa; 2 Center for Reproductive Medicine, Cleveland Clinic, Cleveland, Ohio, USA; 3 Molecular Biotechnology Core Laboratory, The Lerner Research Institute, Cleveland Clinic Foundation, Cleveland, Ohio, USA

## Abstract

**Background:**

Male-factor infertility presents a vexing problem for many reproductively active couples. Many studies have focused on abnormal sperm parameters. Recent advances in proteomic techniques, especially in mass spectrometry, have aided in the study of sperm and more specifically, sperm proteins. The aim of this study was to review the current literature on the various proteomic techniques, and their usefulness in diagnosing sperm dysfunction and potential applications in the clinical setting.

**Methods:**

Review of PubMed database. Key words: spermatozoa, proteomics, protein, proteome, 2D-PAGE, mass spectrometry.

**Results:**

Recently employed proteomic methods, such as two-dimensional polyacrylamide gel electrophoresis, mass spectrometry, and differential in gel electrophoresis, have identified numerous sperm-specific proteins. They also have provided a further understanding of protein function involved in sperm processes and for the differentiation between normal and abnormal states. In addition, studies on the sperm proteome have demonstrated the importance of post-translational modifications, and their ability to bring about physiological changes in sperm function. No longer do researchers believe that in order for them to elucidate the biochemical functions of genes, mere knowledge of the human genome sequence is sufficient. Moreover, a greater understanding of the physiological function of every protein in the tissue-specific proteome is essential in order to unravel the biological display of the human genome.

**Conclusion:**

Recent advances in proteomic techniques have provided insight into sperm function and dysfunction. Several multidimensional separation techniques can be utilized to identify and characterize spermatozoa. Future developments in bioinformatics can further assist researchers in understanding the vast amount of data collected in proteomic studies. Moreover, such advances in proteomics may help to decipher metabolites which can act as biomarkers in the detection of sperm impairments and to potentially develop treatment for infertile couples.

Further comprehensive studies on sperm-specific proteome, mechanisms of protein function and its proteolytic regulation, biomarkers and functional pathways, such as oxidative-stress induced mechanisms, will provide better insight into physiological functions of the spermatozoa. Large-scale proteomic studies using purified protein assays will eventually lead to the development of novel biomarkers that may allow for detection of disease states, genetic abnormalities, and risk factors for male infertility. Ultimately, these biomarkers will allow for a better diagnosis of sperm dysfunction and aid in drug development.

## Background

As infertility affects nearly 15% of all couples of reproductive age [1,2], and family sizes continue to shrink, concerns about the reproductive potential of future generations are growing. Although infertility was believed to originate from female abnormalities, recent discoveries have revealed that as many as 50% of cases stem from male-factor defects, with no identifiable cause in 25% of infertile men [3,4]. Male infertility presents an interesting, yet vexing, problem for men all over the world. Suboptimal sperm quality due to abnormal parameters--motility, morphology, concentration, DNA fragmentation, and genetic composition--has been linked to this issue. Nevertheless, our current understanding of the spermatozoa and its pathological and physiological effects are lacking and vaguely defined.

The study of cellular components on a molecular level offers much hope in deciphering the metabolic pathways essential for the diagnosis of male infertility. Advances in spermatozoa research-including those in the field of proteomics-have allowed for enhanced characterization and identification of both the structural and functional proteins of spermatozoa. However, these findings remain limited. Because the spermatozoon is an extremely complex and highly accessible cell, it is remarkably suitable for proteomic analysis [5]. Moreover, because the sperm cell is responsible for transporting the paternal genome to the oocyte, examining its genetic composition may provide beneficial insight into ensuing disorders in offspring.

Recently employed proteomic techniques, such as 2D polyacrylamide gel electrophoresis (2D-PAGE), mass spectrometry (MS), and differential in gel electrophoresis (DIGE), have allowed for the identification of numerous sperm-specific proteins. These approaches have provided a greater understanding of protein function involved in sperm processes such as motility, capacitation, acrosome reaction, and fertilization. Studies of the sperm proteome have demonstrated how post-translational modifications, such as phosphorylation, glycosylation, proteolytic cleavages and mutations, bring about the physiological changes in spermatozoa function. Furthermore, proteomic analysis has allowed for the study of spermatozoa in different functional states--immature versus mature, uncapacitated versus capacitated, normal versus defective, and low sperm count versus high sperm count--all of which impact the male reproductive potential.

In this review, various proteomic techniques and their usefulness in diagnosing sperm dysfunction, as well as the possible application in the clinical setting will be examined.

### Understanding the need for proteomic research

The human genome was first sequenced 10 years ago [6]. Since then, a vast amount of DNA sequences, including the diploid genome sequence of a human, have been made available in public databases [7]. Investigators are now beginning to understand that the promise of molecular medicine as a cure for genetic diseases was over emphasized, and that merely knowing the human genome sequences is not enough to elucidate the biochemical functions of genes. To unravel the biological display of the human genome, there must be greater understanding of the physiological function of every protein in the tissue-specific proteome. The genetic basis of human disease is much more complex than it was previously thought as a multitude of metabolic and regulatory pathways, post-translational modifications and complex protein-protein interactions play a major role [8]. Therefore organ- and tissue-specific proteomic studies will contribute directly to understand the fundamentals of spermatozoa and its physiological function in male fertility.

Further comprehensive studies on sperm-specific proteome, mechanisms of protein function and its proteolytic regulation, biomarkers and functional pathways, such as oxidative-stress induced mechanisms, will provide better insight into physiological functions of the spermatozoa. Large-scale proteomic studies using purified protein assays will eventually lead to the development of novel biomarkers that may allow for detection of disease states, genetic abnormalities, and risk factors for male infertility. Ultimately, these biomarkers will allow for a better diagnosis of sperm dysfunction and aid in drug development.

### Proteomics*--a brief overview*

During the 1980s there were several new advances in molecular biology such as gene cloning, sequencing, and expression analysis. This transpired to the links between observed activity and protein function and its encoded gene to give way to protein chemistry. These novel approaches of identifying and characterizing proteins by means of both qualitative and quantitative analysis became known as proteomics. Scientists no longer only focused on the genomes of an organism, but rather turned their attention equally towards the structure of proteins and the functional interactions between the proteins.

As research soared in the mid-1990s, scientists attempted to discover a link between the genome expressing the proteins of a living cell and advances in new high-throughput techniques to aid in the process. This interest allowed for the development of successful sequencing analysis, reversible approaches that focused on phenotype instead of genotype, and gene sequencing databases. It was believed that if a link between the genotype and phenotype could be made, it would be plausible to examine all of an organism's proteins instead of concentrating on one to further understand their structure, function and their biochemical role in the body.

Proteomic approaches are generally now utilized for protein profiling, comparing the protein expression in specific organs and tissues, localizing and identifying post-translational modifications, and studying protein-protein interactions. New fractionation and labeling techniques have further enhanced protein identification of some of the least abundant proteins. It is these less prevalent proteins that have major impacts on biological systems and may be potentially valuable in clinical diagnosis and prescribing treatment.

Although the total number of human protein products has been estimated to be approximately one million, and comparison of different proteomes is possible via analysis of post-translational modifications, there still remain several limitations and hurdles [9-11]. The proteomes of mammalian cells, tissues, and bodily fluids, and their readily active concentrations and local environment are so intricate that even new techniques are unable to make fully accurate measurements. The large quantity of data that has been obtained thus far places further challenges on data processing and analysis. Nevertheless, while researchers now face additional limitations in fully sequencing proteins and peptides due to the complex nature and physiological processes of the proteome, extensive advancements in technology, methods of analysis and ways to overcome these matters hold for a promising future.

### The basic process

In order to understand how human seminal proteins can be identified and characterized, the basic process of proteomic analysis will be described in brief. After the collection and liquefaction of a neat semen sample, it can be used as is or further processed (e.g. separating into mature and immature sperm populations by density gradient centrifugation). The recovered spermatozoa can be extracted and purified, after which the protein concentration can be determined in order to allow for equal protein-loading gradient on electrophoretic gels. Because proteins differ from each other in terms of their charge and mass, they can be separated using gel electrophoresis methods, such as one-dimensional sodium dodecyl sulfate polyacrylamide gel electrophoresis (1D SDS-PAGE), two-dimensional sodium dodecyl sulfate polyacrylamide gel electrophoresis (2D SDS-PAGE) or DIGE.

Various stains (e.g. Coomassie Brilliant Blue and Silver) are used to identify the positions of individual proteins within the gel, which appear as spots or smudges. These in turn can be excised and analyzed by MS. Peptides generated by in-gel digestion of the proteins in the 1D/2D SDS-PAGE gels and those digested by trypsin in DIGE samples can be extracted and subsequently purified by liquid chromatography. The extracted peptides can be directly analyzed by matrix assisted laser desorption ionization-time of flight (MALDI-TOF-TOF) or liquid chromatography - mass spectrometry/mass spectrometry (LC-MS/MS). These purified peptides can then be subjected to MS, or tandem MS (MS/MS), to identify the protein by peptide mass mapping as well as by sequencing the peptides by further fragmentation characteristic mass-to-charge ratio (m/z) of ions that is resembled in a specific mass spectrum. Finally, the data obtained can be analyzed, and the amino acid sequence can be submitted into a database to search for matching peptide sequences and thereby identifying the protein(s) that are most likely present.

### Techniques

The initial purpose of proteomics was simply to analyze a proteome--the protein compliment expressed by a genome--using techniques such as two-dimensional gel electrophoresis (2D-E) and protein staining followed by in-gel digestion, peptide extraction and identification of peptide protein spots using MALDI-TOF-TOF or MS/MS [12-16]. The highly dynamic proteome depends on environmental conditions as the proteins' activity and their abundance vary at different physiological states and locations of the cell or tissue. Over time, the field expanded into profiling functional and structural proteomics, specifically aiming to identify and characterize a complete set of proteins present in a cell, organ, or organism [12]. In order to accommodate this higher level of focus, a wide range of techniques has been employed.

Profiling proteomics attempts to differentiate proteins expressed between two samples (cells or organisms) while exemplifying the variations in expression levels of two different states [17]. Distinguishing alternations in the proteome during normal or stressed conditions may help elucidate the processes of signaling pathways in certain disease states. On the other hand, functional proteomics is employed when searching for protein functions on post-translationally modified proteins, which are essential in understanding the functions and role of protein in a living organism [17]. Finally, structural proteomics focuses on the tertiary structure of proteins and their complexes with other small molecules and proteins [17]. Understanding how proteins interact with their corresponding binding substrates may help uncover the mechanistic pathways these molecules undergo and subsequently further improve our knowledge of the proteome and its relation to human life adversities.

**Figure 1:**
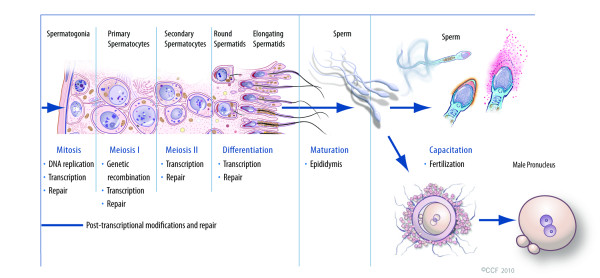
**Cellular changes occurring during spermatogenesis**.

Many researchers have begun to note the abundance of proteins and their functions in humans. The human genome harbors 31000 protein-encoding genes [18]. New advances in proteomics have contributed to the most recent knowledge and understanding of how spermatozoa function and acquire the ability to fertilize [19]. Post-translational modifications (PTM) are essential for the functionality of spermatozoa, both during the maturation of the cells in the epididymis and post-ejaculatory capacitation in the female reproductive tract (See Figure 1) [19]. The modifications that are made via glycosylation, methylation, or phosphorylation are capable of altering the functional properties of spermatozoa and seminal plasma proteins that consequently result in abnormal and difficult to detect effects. Therefore, understanding the function of each protein requires information not only on quantitative levels of gene expression at both the mRNA (transcriptome) and protein (proteome) levels but also on quantitative levels of proteolytic activity and eventually information on endogenous protein substrates and products along with their physiological significance.

**Figure 2:**
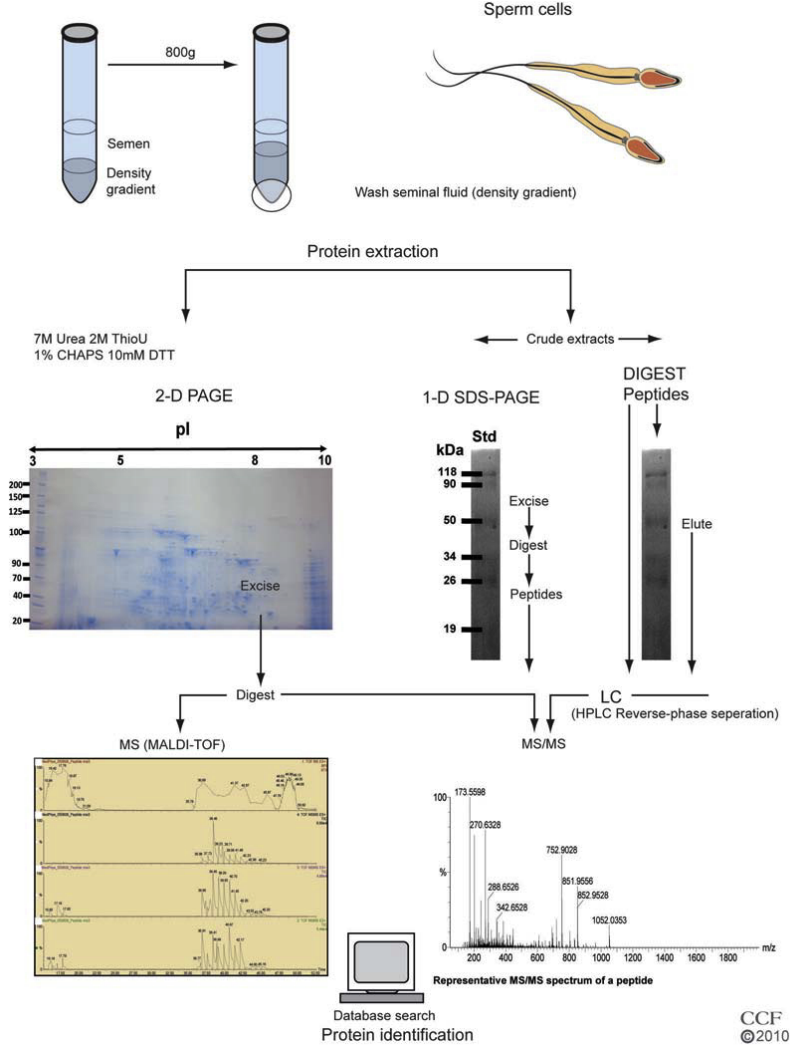
**Extraction and analysis process of proteins from spermatozoa**.

As the field of proteomics continues to grow, advances in technology and techniques have allowed for enhanced protein separation, fractionation, purification, detection, isolation, identification and characterization. Most of the recent proteomic studies have focused on improving the resolution of separation and the identification of every protein by gel electrophoresis and MS analysis [15]. In addition, peak and multidimensional separations have given us further insight into a protein sample while also elevating the sensitivity for sample analysis [20-23]. Nevertheless, separation of a complex mixture of protein and peptides still remains one of the most difficult challenges. If it is possible to quantitatively identify uniquely expressed proteins in a disease state (as a biomarker) then a link to possible disease states can be established and potential therapeutic treatments may be discovered. The following subsections will give an overview of some of the most commonly used proteomic techniques that have allowed for the extraordinary advances in the field, particularly in sperm cell biology (See Figure 2).

#### Gel electrophoresis

Gel electrophoresis is a common gel-based technique that has played a revolutionary role in the field of protein separation studies. This method separates deoxyribonucleic acid (DNA), ribonucleic acid (RNA), and proteins from complex mixtures, allowing for the detection and approximate quantification of proteins. In general, electrophoresis utilizes an applied electromotive force to a cross-linked polymer gel matrix that can limit the motion of molecules based on their mass and charge. Since 1D gel electrophoresis methods have limited resolving power to separate complex mixtures of proteins, particularly low-abundance proteins, multidimensional approaches have been currently employed to overcome these challenges.

### Two-dimensional gel electrophoresis

2D-E is a widely accepted electrophoresis method that analyzes qualitative and quantitative characterization of proteins and changes in the proteome at high resolution on a large scale. It can thus serve as an initial screening method to acquire hypotheses and establish future explorative directions. The proteins on the gel may represent a compliment of active proteins in a particular tissue or cell at any given time.

In addition, the resolving power and sensitivity as well as the low equipment cost are attractive to researchers. However, heterogeneities in different gels, the electric fields, pH gradients, and thermal fluctuations are only a few factors that may influence enzymatic activity, thereby making protein verification difficult. Therefore, much focus has been given to improving methods to overcome these variations and detect low-abundance protein modifications, which are the primary proteins of interest. This would give us a better understanding of protein structure and function as well as provide a plausible diagnostic tool.

2D-PAGE examines proteins based on their isoelectric point (pI) via isoelectric focusing separation in the first dimension followed by separation in the second dimension based on molecular mass using sodium dodecyl sulfate polyacrylamide gel electrophoresis (SDS-PAGE). SDS-PAGE specifically separates proteins according to their electrophoretic mobility--a function of polypeptide chain length or molecular mass. Polyacrylamide provides a support matrix through which proteins can migrate. Since 2D-PAGE is not sensitive enough to detect rare or low-abundance proteins, many proteins will not be resolved, and further splitting of the sample into different subcellular fractions may be necessary to reduce the complexity of protein mixtures prior to 2D-PAGE analysis.

Proteins have varying charges, shapes and sizes and thus travel at different rates through the gel. To address this problem, proteins are usually denatured in a detergent, such as SDS, which coats the protein with a negative charge. In turn, the charge of the protein no longer becomes a contributing factor to its movement on the gel, and protein size becomes the main determinant. In other words, the larger the protein, the more restricted its movement will be towards the positively charged cathode. The protein's molecular mass is then compared to a standard ladder, which is loaded in a separate lane. Upon staining, a visible spot indicates the presence of a protein or possibly a multitude of proteins. Proteins can be then identified through qualitative analysis.

Although 2D-E-based studies have generated great insight on the overall nature and complexity of the sperm proteome in terms of iso-electric point and molecular mass, precise protein identification and characterization remains a challenge with this approach. 2D-PAGE is a labor-intensive technique that is susceptible to gel matrix irregularities due to experimental conditions such as laboratory humidity, voltage fluctuations, and preparation slip-ups. Alternative methods can be used to circumvent these challenges: MS and two-dimensional difference in gel electrophoresis (2D-DIGE).

### Mass spectrometry

MS is an analytical tool that identifies proteins or peptides by measuring the masses of molecules converted into ions via their m/z ratio. It is even capable of determining the elemental composition of a molecule. In addition, MS can be clinically useful in profiling biomarkers from tissues or bodily fluids as a method to diagnose different medical conditions.

Two of the most common MS techniques that have been applied to study sperm proteins are MALDI-TOF MS and LC-MS/MS. Both can account for systematic biases when characterizing protein spots from a previously-run-electrophoresis gel. The spectral data obtained from MS gives the protein's m/z ratio and its associated intensity, which aids in determining protein differential expression and modifications.

With MALDI-TOF analysis, it is possible to determine a protein's m/z ratio by excising protein bands from the gel and subsequently digesting them with trypsin. After the peptide masses or peptide fingerprints have been recorded, they can then be matched to sets of theoretically digested protein reference masses from a database to identify the protein (peptide mass mapping).

On the other hand, LC-MS/MS is a highly sensitive and specific analytical technique that combines the physical separation of peptides generated by trypsin digestion of a spot by HPLC (high performance liquid chromatography) followed by mass determination and subsequent sequence in MS/MS set up. The use of HPLC prior to MS has fueled tremendous growth in proteomics over the past few years. Its initial application for protein or peptide separation is based on the number of unique properties such as charge, hydrophobicity and the presence of a specific tag or amino acid(s) [17]. The subsequent coupling to a tandem mass spectrometer with an interface allows for rapid separation and identification of the complex protein mixtures and in turn provides the primary peptide sequence of the original complex protein.

Both 2D separation-based MALDI-TOF and LC-MS/MS methods can be reproduced in laboratories and provide a 2D-map of the proteins in a complex mixture based on their iso-electric point and molecular mass. Since MALDI-TOF MS can be less sensitive and time consuming, advancements in LC-MS/MS analysis provide an enhanced method for protein identification throughput and a potential future diagnostic tool.

Nevertheless, MS is not perfect. "Noisy" data from external interference may give false positive peaks, making it difficult to detect protein changes, especially in low-copy and low-abundance proteins. Alternatively, tandem mass spectrometry (MS/MS) can be applied to further fragment protein mixtures, and more importantly, to improve the detection limits of compounds and the signal-to-noise ratio relative MS. However, the total ion current of some compounds may be decreased using MS/MS while also possibly affecting the efficiency, reproducibility and detection of the mass spectra.

### Difference in gel electrophoresis

DIGE is a type of gel electrophoresis used to detect quantitative changes in protein abundance. This technique has played an essential role in the advancement of proteomics in that it takes the focus off the whole protein and places it onto its constituent peptides [19]. It is at the protein level where most PTMs occur that cause the significant variations in the three-dimensional protein structure and gel electrophoretic behavior. Thus, it is the peptides that provide the best basis of proteomic comparison [19]. DIGE can overcome 2D-E limitations that are due to inter-gel variation by including an internal standard in each gel and also provide faster throughput for subsequent analysis-since the proteins from the different sample types are run on the same gel, they can be directly compared. Therefore, this technique has the potential to be used one day in a clinical setting to compare samples of healthy and diseased states to formulate a prognosis for future direction and treatment.

#### Bioinformatics

One of the most daunting challenges currently facing proteomics is making sense of the vast data collected thus far. Bioinformatics, which employs statistical analysis and algorithms, can assist proteomic-based studies in analyzing data and identifying proteins of interest from MS or other technical data [24]. One system used by bioinformatics is Gene Ontology (GO), which can reveal meaningful patterns found in proteins [25]. A recent study using fly models employed GO, leading to the development of new ideas on gene regulation and chromatin organization [26]. Proteomics will expand on the current knowledge of human spermatozoa as bioinformatics applications continue to be developed in the future.

#### Future direction of techniques

Due to the complexity of proteomic samples, 1D separation techniques are no longer sufficient. Because PTMs occur at the peptide level-and they are the main factors that distinguish similar samples-additional multidimensional separation techniques must be employed to reduce a protein's complex nature. Advances in chromatographic columns based on restricted access materials and monolithic columns have been suggested to improve selectivity. In addition, stationary phases have been introduced to improve the enrichment of low-abundance proteins and their detection. Isotope-coded affinity tag (ICAT) and isobaric tag for relative and absolute quantification (iTRAQ) are recently developed proteomic methods used to identify and quantify low-abundance and low-concentration proteins. Nevertheless, advancements in MS must keep up with the fast-developing multidimensional separation techniques to achieve optimum resolution.

Furthermore, both consistent analysis of LC-MS/MS and sample throughput have been a few of the key bottlenecks for many proteomic studies [27]. LC-MS/MS data requires multiple stages of analysis, and protein separation often takes several hours to separate a single sample. A proposed approach to improve sample throughput is to develop faster scanning mass spectrometers. In order to overcome some of the most common challenges in the field, it is necessary to improve the effectiveness of sample preparation to reduce the protein's complexity and to enrich the low abundance proteins while decreasing the more abundant, less significant ones. Miniaturization and extensive data processing and analysis are also important.

### Proteomics and mature spermatozoa

Until recently, most proteomic analysis of spermatozoa was largely conducted utilizing the 2D-PAGE approach. In terms of molecular mass and iso-electric point, 2D-E-based studies have been valuable in generating data on the overall nature and complexity of the sperm proteome and the nature of some of those proteins targeted by the human immune system. An advanced 2D-PAGE resolution using narrow pH ranges for iso-electrofocusing reported 3,872 different protein spots--16 proteins of significance were identified [28].

Early sperm proteome analyses identified 1397 protein spots--at least 98 surface-related--and were catalogued by means of 2D-PAGE analysis [29]. An *in vitro *experiment conducted with prolactin, one of the tryrosine kinase surface receptors, demonstrated improved fertilizing potential of mammalian spermatozoa after the addition of the hormone [30]. Earlier studies have noted insulin to be a hormone of interest from its displayed interaction with both the plasma membrane and sperm acrosome [31]. Moreover, these reports further reinforced the essential role of surface receptors during fertilization, and any defect or abnormality may result in suboptimal reproductive potential or even infertility. Additional proteomic analysis at the basal level of the sperm's surface can provide additional knowledge and insight into the possible biological mechanistic processes involved in acrosome reaction, capacitation, and ultimately, fertilization.

**Figure 3:**
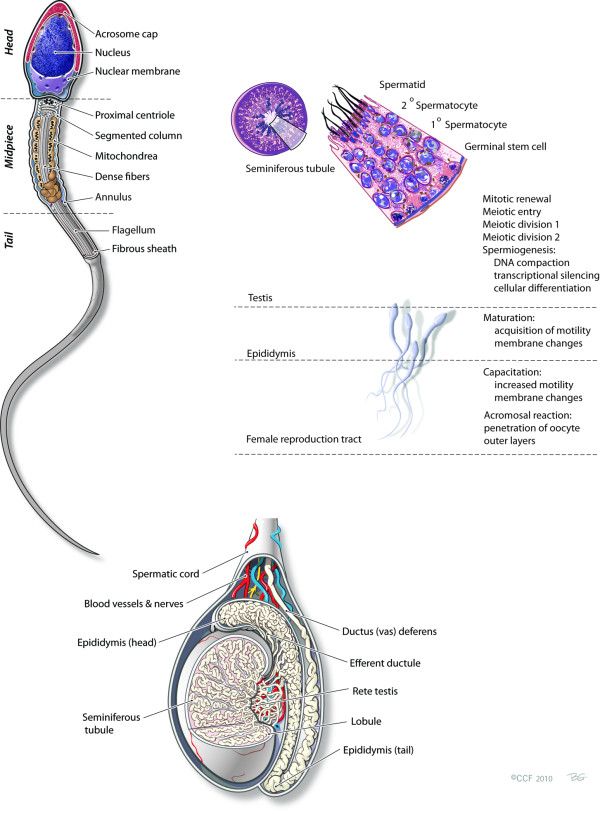
**Human sperm and its formation and associated processes**.

Furthermore, several proteomic studies (Refer to Table 1) have uncovered numerous housekeeping sperm proteins involved in fundamental processes such as oxidative phosphorylation and glycolysis [28,29,32-40]. Aitken *et al*. (1998) discussed the indispensable role of NADPH oxidases in sperm capacitation to help drive cAMP-induced tyrosine phoshorylation signal transduction cascades through possible suppression of tyrosine phosphatase activity [41]. Inhibiting protein kinase A (PKA) phosphorylation of A-kinase anchor proteins (AKAPs) revealed hyperactivation impairment, resulting in suboptimal reproductive function and subsequent infertility [38,41-43]. Human spermatozoa have been found to contain all the machinery necessary to sustain a cAMP-signaling pathway in the sperm tail and the components associated with the 26S proteasome [29,37,44]. The 26S proteasome is an ATP-dependent proteolytic complex essential in the sperm-egg interaction [29,37,44]. Indeed, past proteomic studies hold for a promising future of discovering new sperm proteins, which in turn will allow researchers and clinicians to better understand and diagnose sperm dysfunction (See Figure 3).

**Table 1:** Proteomic studies on mature human spermatozoa

Authors	Study	Proteomic Technique	Outcome
Johnston et al. 2005 [37]	First extensive analysis of human sperm proteome	LC-MS/MS	1760 proteins identified
Martinez-Heredia et al. 2006 [36] de Mateo et al. 2007 [40]	Goal to provide a reference 2D map of the mature human sperm proteome	MALDI-TOF	131 proteins identified
Ficarro et al. 2003 [38]	Phosphoproteome analysis of capacitated human sperm	LC-MS/MS	60 proteins identified
Domagala et al. 2007 [32]	Identification of sperm immunogenic antigens	LC-MS	35 proteins identified
Secciani et al. 2009 [33]	Protein profile of capacitated versus ejaculated human sperm	MS/MS	116 proteins identified
Shetty et al. 2001 [39]	Identification of immunocontraceptive candidates	MALDI-MS	8 proteins identified
Bohring et al. 2001 [34]	Isolation and identification of sperm membrane antigens	MALDI-MS	18 proteins identified
Baker et al. 2007 [29]	Identification of proteins in human sperm using LC-MS/MS analysis	LC-MS/MS	1056 proteins identified
Lefievre et al. 2007 [35]	Identify targets for S-nitrosylation in human sperm	LC-MS/MS	240 proteins identified
Li et al. 2007 [28]	Establish high-resolution 2-D map of human sperm proteins	MALDI-MS	16 proteins identified

Comparative studies employing proteomic techniques have also helped to identify proteins of interest in infertile men in comparison to fertile men. Several comparative studies have recently been completed, most notably a 2007 study performed by Zhao *et al*. [45]. The study compared 8 men with asthenozoospermia with healthy controls. The authors identified 10 of 17 proteins with either increased or decreased frequency in the asthenozoospermic samples. They were able to link these differences to enzymes involved in sperm metabolism. Such comparative studies are expected to identify biomarkers, which will aid clinicians in better diagnosing male-factor infertility.

Proteomic approaches are now focused on characterizing the tyrosine phosphorylated proteins to better understand and clarify the mechanistic processes behind the capacitation-dependent alternations involved in sperm function. In the future, with improvements in proteomic methods, it will be possible to generate an accurate proteomic profile of normal human spermatozoa, which can aid in understanding and differentiating between the various functional stages of sperm cells (e.g., mature versus immature, capacitated versus uncapacitated, and normal versus defective parameters). With this information, it would be possible to verify whether impaired sperm function is related to PTMs involved in generating normal functional spermatozoa. Further understanding of various physiological and biochemical changes in post-testicular sperm maturation will unravel the molecular basis of defective sperm function characterized in subfertile men.

A study by Shetty *et al*. identified several novel sperm protein sequences from a 2D-E gel [39]. Many of those detected shared identical sequences, further stressing the importance of PTMs to sperm proteins. Another detailed MALDI-TOF analysis of the human sperm proteome consisting of over 100 mapped spots on 2D gels by Martinez-Heredia *et al*. identified 98 different proteins, and 23% of them had yet to be recorded on the human spermatozoa [36]. In addition, proteomics is a continuously evolving field and researchers are constantly exploring new avenues to further define and characterize the human proteome. The recent application of a highly sensitive nanoscale liquid chromatography technique combined with MS identification yielded 1056 gene products, 8% of which had not been previously characterized [29].

### Clinical applications of proteomics

Although proteomics has only been used to study human spermatozoa in the laboratory setting, proteomic-based studies may one day be used in the clinical setting to identify sperm dysfunction. Sperm chromatin, which consists of DNA and proteins, is essential for proper sperm function and embryonic development, as several studies have linked defects in sperm chromatin to potential reproductive errors [46-48]. Proteomics offers the potential for expanding on clinical diagnostic testing of sperm for infertility, which is currently limited to count, motility and morphology.

In order to apply proteomics to a clinical setting, several areas of study must be expanded upon. The first milestone that must be reached in order to better understand human spermatozoa is to determine the entire human sperm proteome. While a study by Baker and colleagues identified 1056 proteins and a more recent study by Li and colleagues identified 3872 spots, very little is known about the sperm proteome [28,29]. Prior studies have already been conducted on seminal plasma. Therefore, once more studies are completed, a better understanding of human ejaculate will be gained [49]. Future proteomics studies will allow for a better understanding of PTMs such as phosophorylation and sperm capacitation [38,50]. These PTMs are vital for consequent stages of sperm formation, maturation and activation [51]. Currently, there is a very limited understanding of these modifications, but this area of study is continuing to expand in scope with the pending publication of new studies.

A second milestone is the publication of comprehensive comparative studies between normal spermatozoa and abnormal spermatozoa. Such studies will highlight the potential of proteomics to identify biomarkers that can, in turn, help clinicians pinpoint certain peptides or metabolites that may be linked to male infertility. Furthermore, proteomics can be employed in comparative studies between human sperm samples and model organisms such as mice, fish, worms, flies, and sea urchins, all of which have already contributed to greater understanding of human spermatozoa [5,52].

Protamines present a starting point for proteomic studies to identify proteins with altered expression in infertile patients. For example, one recent study by de Mateo and colleagues found an altered ratio of two protamine proteins-protamine 1 and protamine 2, also known as P1 and P2, respectively [40]. In addition, proteomic studies using mouse models have identified the chaperone protein HSPA2 as an important assessment factor for infertility in humans [5]. One study using asthenozoospermic patients identified HSPA2 as one of 17 proteins with abnormal levels in comparison to samples from normal donors [53]. Since HSPA2 is believed to be vital to several sperm processes such as plasma membrane remodeling and cytoplasmic extrusion, HSPA2 resembles a biomarker that can aid in clinical diagnosis of male infertility (e.g., less or absent sperm-specific proteins may correspond to incompletely formed or defective sperm chromatin) [54].

Studies such as these can produce vast amounts of data, but with the help of bioinformatics applications, researchers can discover meaningful patterns that may be applied in a clinical setting [55]. Furthermore, similar proteomic studies may identify additional biomarkers that are indicative of male-factor infertility signaling pathways.

### Factors affecting proteomic studies

Several factors affect proteomics, all of which must be taken into account when putting proteomics into practice in the clinical setting. The 2D-PAGE technique is laborious and indefinite in identifying and characterizing proteins [5]. In a study by Li *et al.*, only 16 of 3872 spots were identified using this technique, while in similar experiment conducted by Martinez-Heredia *et al*., only 98 of 1000 spots were identified [28,53].

Variations in study findings can be attributed to numerous factors, many of which are uncontrollable in current proteomic advances. Pre-analytical variables may affect the testing for biomarkers during sample collection, pipetting, and dilution [55]. Human biological variation with age, race, nutrition, life style, stress, environment or global location of the sample population may also affect experimental results [56].

Nevertheless, biomarker discovery remains a very challenging task due to the complexity of the sample as well as the wide dynamic range of protein concentrations [24]. As the field of proteomics emerges to face several challenges, bioinformatics can provide statistical support in analyzing the vast amounts of data resulting from proteomics testing. Although proteomics is an emerging field facing many challenges, bioinformatics can provide statistical support in analyzing the vast amounts of data resulting from proteomics testing. However, it is expected that the future proteomics studies may lead to the development of novel diagnostic tools and therapeutic drugs to improve sperm dysfunction by elucidating potential causes of sperm impairment and providing insights to its underlying mechanistic pathway [50].

## Conclusion

As researchers and scientists note the sudden decline in male reproductive potential worldwide, many have focused their attention on male-factor defects. Several reports have linked suboptimal sperm quality to this growing concern [57,58]. Although much remains unknown about human spermatozoa and its pathological and physiological effects on reproductive function, recent advances in proteomic techniques have provided insight into sperm function and dysfunction. Several multidimensional separation methods have been utilized to identify and characterize spermatozoa [28,29,37]. Future developments in bioinformatics can further assist researchers in understanding the vast amount of data collected in proteomic studies. In addition, such advances in proteomics may help to decipher metabolites directly involved in normal growth, development, and reproduction, which can stand as biomarkers in the detection of disease or sperm impairments. Multinuclear nuclear magnetic resonance-based metabolomic approaches along with advanced proteomic techniques may provide essential information of the cellular metabolome in order to further understand cellular function. With the identification of novel biomarkers through proteomic studies, clinical tests and treatments for sperm dysfunction might be developed to potentially help infertile couples.

## Competing interests

The authors declare that they have no competing interests.

## Authors' contributions

SSDP conceived the study, contributed to the design of the study, information collection, drafting of the manuscript and critical review of the final paper. AK helped with the design of the study, information collection and drafting of the manuscript. DB helped with information collection and drafting of the manuscript. AA and SY have been involved in the critical revision of the manuscript. All authors read and approved the final manuscript.

## Authors' information

Stefan S du Plessis, PhD, is the head of the Division of Medical Physiology and associate-professor in the Faculty of health Sciences at Stellenbosch University, Tygerberg, South Africa. Anthony H Kashou and David J Benjamin are summer interns at the Center for Reproductive Medicine, Cleveland Clinic, Cleveland, Ohio, USA. Satya Yadav is the director of the Molecular Biotechnology Core Laboratory of The Lerner Research Institute at the Cleveland Clinic Foundation, Cleveland, Ohio, USA. Ashok Agarwal is the Director of the Center for Reproductive Medicine, Glickman Urological & Kidney Institute and Ob/Gyn & Women's Health Institute, Cleveland Clinic, Cleveland, Ohio, USA
